# Effect of 50 Hz Extremely Low-Frequency Electromagnetic Fields on the DNA Methylation and DNA Methyltransferases in Mouse Spermatocyte-Derived Cell Line GC-2

**DOI:** 10.1155/2015/237183

**Published:** 2015-08-03

**Authors:** Yong Liu, Wen-bin Liu, Kai-jun Liu, Lin Ao, Julia Li Zhong, Jia Cao, Jin-yi Liu

**Affiliations:** ^1^College of Bioengineering, Chongqing University, Chongqing 400044, China; ^2^Institute of Toxicology, College of Preventive Medicine, Third Military Medical University, Chongqing 400038, China

## Abstract

Previous studies have shown that the male reproductive system is one of the most sensitive organs to electromagnetic radiation. However, the biological effects and molecular mechanism are largely unclear. Our study was designed to elucidate the epigenetic effects of 50 Hz ELF-EMF *in vitro*. Mouse spermatocyte-derived GC-2 cell line was exposed to 50 Hz ELF-EMF (5 min on and 10 min off) at magnetic field intensity of 1 mT, 2 mT, and 3 mT with an intermittent exposure for 72 h. We found that 50 Hz ELF-EMF exposure decreased genome-wide methylation at 1 mT, but global methylation was higher at 3 mT compared with the controls. The expression of DNMT1 and DNMT3b was decreased at 1 mT, and 50 Hz ELF-EMF can increase the expression of DNMT1 and DNMT3b of GC-2 cells at 3 mT. However, 50 Hz ELF-EMF had little influence on the expression of DNMT3a. Then, we established DNA methylation and gene expression profiling and validated some genes with aberrant DNA methylation and expression at different intensity of 50 Hz ELF-EMF. These results suggest that the alterations of genome-wide methylation and DNMTs expression may play an important role in the biological effects of 50 Hz ELF-EMF exposure.

## 1. Introduction

With the application of a number of electrical devices, living and working environments are subject to different levels of extremely low-frequency electromagnetic fields (ELF-EMFs). In recent years, the growing professionals and the public have dramatically concerned about the potential hazardous effects of ELF-EMF exposure on human being. Many studies have reported that human health is associated with the exposure to ELF-EMF, especially focusing on a variety of diseases, including children leukemia, brain cancer, Lou Gehrig's disease, and Alzheimer's disease [1–8]. The male reproductive system is one of the most sensitive organs to electromagnetic radiation. Although adverse effects of ELF-EMF on human male reproduction are still controversial, substantial evidence indicates that exposure to ELF-EMF can decrease human and animals semen quality by reducing the motility and increasing oxidative stress and morphometric abnormalities of spermatozoa [9–14]. In spite of numerous attempts, the biological effects and its mechanism of ELF-EMF remain unclear.

ELF-EMF exposure was considered genotoxicity although the energy transferred into the cells was not high enough to cause direct damage to DNA. However, it may have indirect effects on the DNA structure. Epigenetics is the heritable and stable changes of gene expression or cellular phenotype without DNA sequence alterations, including DNA methylation, noncoding RNAs, and histone modifications [15, 16]. In recent years, many researches showed that epigenetics is closely related to embryonic development, autoimmune diseases, cancer, and central nervous system diseases [17, 18].

DNA methylation occurs at the cytosine residues of CpG dinucleotides by an enzymatic reaction that produces 5-methycytosine (5-mC), which is catalyzed by DNA methyltransferases (DNMTs). It is an extensively characterized mechanism for epigenetic regulation and plays an important role in the regulation of gene expression [19]. Aberrant hypermethylation of CpG islands in gene promoter regions frequently occurs in human cancers. In higher eukaryotes, three active DNMTs have been identified. DNMT1 is involved in the maintenance of methylation pattern that copies the preexisting methylation patterns onto the newly replicated strand during DNA replication, while DNMT3 is involved in de novo methylation for methylating previously unmethylated CpG sites and is encoded by 2 distinct genes, DNMT3a and DNMT3b. As the main enzyme for DNA methylation, DNMTs is not only associated with DNA methylation, but also links to many important biological activities, including cell proliferation, senescence, and cancer development.

Previous studies have reported that DNA methylation is an important regulator of gene transcription and a large body of evidence has demonstrated that aberrant DNA methylation is associated with unscheduled gene silencing, and the genes with high levels of 5-methylcytosine in their promoter region are transcriptionally silent [20, 21]. Therefore, there is the possibility that ELF-EMF exposure could cause epigenetic modification changes in cells, and this may account for the adverse effects of ELF-EMF on the male reproductive system. In order to investigate a plausible mechanism, we exposed mouse spermatocyte-derived GC-2 cells to 50 Hz ELF-EMF exposure at an intensity of 1 mT, 2 mT, and 3 mT for 72 h. We observed whether 50 Hz ELF-EMF exposure can induce the alterations of genome-wide methylation and the expression of DNMTs in GC-2 cells.

## 2. Materials and Methods

### 2.1. Cell Culture

Mouse spermatocyte-derived GC-2 cell line was obtained from the American Tissue Culture Collection (ATCC, Rockville, MD, USA) and cultured in DMEM high glucose medium (HyClone, Logan, UT, USA) containing 10% fetal bovine serum at 37°C in a humidified atmosphere with 5% CO_2_.

### 2.2. Exposure Procedure

GC-2 cells were plated onto 90 mm Petri dishes at a density of 4 × 10^5^ cells/5 mL. The ELF-EMF exposure system used in our experiments is the EXF system (Zurich, Switzerland). This EXF exposure system is designed for the testing of ELF-EMF exposure in the 50 Hz frequency range such as alternating current transmission facilities. The exposure system was composed of a power frequency generator, an arbitrary function generator, a narrow band amplifier, and two rectangular waveguides. Exposed and sham-exposed cell dishes were simultaneously placed into an incubator in which the environmental conditions were constant (37°C, 5% CO_2_). The setup generated a vertical EMF, which is composed of two four-coil systems (two coils with 56 windings, two coils with 50 windings), and was placed inside a metal chamber. During the exposure, the value of magnetic field was monitored and the temperature of the medium was assessed as described previously [22]. The temperature difference between sham and ELF-EMF exposure never exceeded 0.3°C. The entire equipment was controlled by a computer, which can automatically control the exposure parameters including exposure intensity and exposure time. After starvation (cultured with serum-free medium overnight), GC-2 cells were exposed to 50 Hz ELF-EMF at magnetic field intensity of 1 mT, 2 mT, and 3 mT with an intermittent exposure (5 min on and 10 min off) for 72 h. The cultured cells were designed to two groups: one is sham control; the other is 50 Hz ELF-EMF exposure. Cells were collected after 72 h of exposure.

### 2.3. Analysis of Global DNA Methylation

The total genomic DNA was extracted from the GC-2 cells using the DNA isolation Kit (Promega, Madison, WI, USA) according to the manufacturer's instructions. The genome-wide methylation level was detected by DNA Methylation Quantification Kit (Epigentek, New York, NY, USA) following the manufacturer's protocol. The analysis provides the levels of global DNA methylation, and it is not specific to any particular gene. The data were presented in terms of percent of control (sham-exposed GC-2 cells). Experiments were carried out in triplicate.

### 2.4. RNA Extraction and Real-Time PCR

Total RNA was extracted from the GC-2 cells using Trizol Reagent Kit (Invitrogen, Carlsbad, CA, USA). Complementary DNA was synthesized using 2 *μ*g RNA through the reverse transcription reaction by PrimeScript RT reagent kit with gDNA Eraser (Takara, Otsu, Japan). The mRNA expression of DNMT1, DNMT3a, and DNMT3b was measured by real-time PCR. The real-time PCR was performed with the IQ5 Real-Time PCR Detection System (Bio-Rad, Hercules, CA, USA), using the SYBR Green I detection method. Each real-time PCR reaction (20 *μ*L total volume) contained 2 *μ*L of cDNA, 10 *μ*L of 2 × SYBR Green Real-time PCR Master (Takara), 0.5 *μ*L of each of the forward and reverse primers, and 7 *μ*L ultrapure water. Samples were denatured by heating at 95°C for 30 sec, followed by 40 cycles of amplification (95°C for 5 sec and 60°C for 30 sec). The mRNA expression levels of DNMTs were normalized to the expression level of the *β*-actin mRNA in each sample using the cycle threshold (Ct) method and the 2^−ΔCt^ formula. Each measurement was repeated for three times and was normalized against the control group.

### 2.5. Western Blot Analysis

Lysates of GC-2 cells were prepared for Western blot analysis. Proteins (40–60 *μ*g) were resolved onto 10% sodium polyacrylamide gel electrophoresis and transferred onto nitrocellulose membranes (Bio-Rad, USA). The membranes were incubated in blocking buffer for 2 hours at room temperature and then incubated with the primary antibodies of DNMT1, DNMT3a, and DNMT3b (Santa Cruz Biotechnology Inc., CA, USA) in antibody dilution buffer overnight at 4°C. The membrane was then washed with phosphate buffered saline for 3–5 times and incubated with HRP-conjugated secondary antibody (Beyotime, Shanghai, China). The enhanced chemiluminescence kit (Beyotime) was used for the detection of signals. Equal loading of proteins on the gel was verified by reprobing the membrane with *β*-actin antibody.

### 2.6. Genome-Wide DNA Methylation Analysis and Validation

The potential mechanism was investigated in detail using Affymetrix microarrays analysis. In brief, the methylation of genes promoter were analyzed using Affymetrix Mouse Promoter 1.0R Array. Gene expression was analyzed by the Affymetrix Mouse Gene 2.0 ST microarrays. Microarray experiments and data analyses were carried out at the Gminix company (Shanghai, China) as reported previously [23]. Some genes were selected according to the highest fold change and verified with methylation-specific PCR (MSP) and real-time PCR.

### 2.7. Bisulfite Conversion and MSP

The DNA was extracted from the GC-2 cells using the DNA isolation kit following the manufacturer's instructions. DNA was bisulfite converted EZ DNA Methylation-Gold Kit (Zymo Research, Orange, CA, USA) according to the manufacturer's instruction. Primer pairs that specifically amplified either methylated or unmethylated sequences spanning the CpG island of these genes were used for MSP as detailed in Table S1 (see Supplementary Material available online at http://dx.doi.org/10.1155/2015/237183). PCR conditions for MSP have been standardized in our laboratory [24, 25].

### 2.8. Statistical Analysis

All the experimental data were expressed as the means ± SD. The statistical significance of difference between the values of sham and exposure was determined using analysis of variance. *P* < 0.05 was considered as statistically significant.

## 3. Results

### 3.1. Effect of 50 Hz ELF-EMF on Genome Methylation Level in the GC-2 Cells

**Figure 1:**
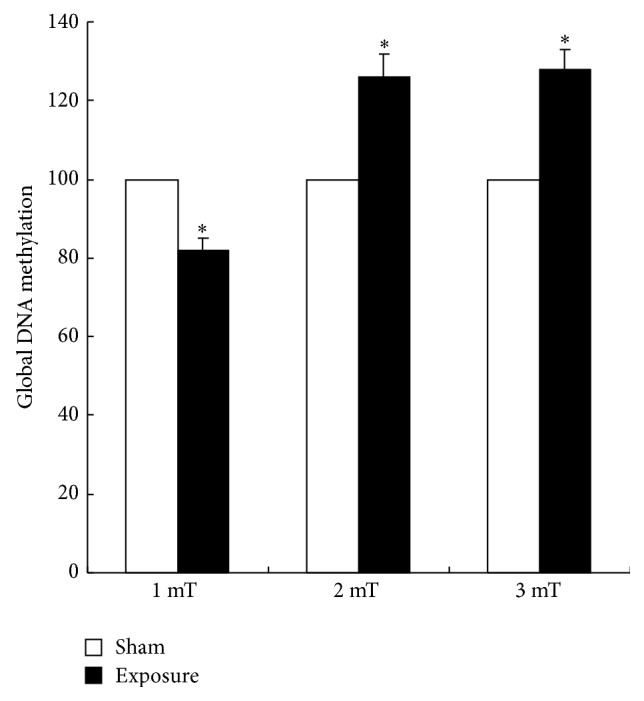
Quantitative analysis of global DNA methylation in GC-2 cells. DNA methylation in GC-2 cells was lower than the sham-exposure group at magnetic field intensity of 1 mT and was higher than the sham-exposure group at 2 mT and 3 mT exposure to 50 Hz ELF-EMF.

As male reproductive system is one of the most sensitive organs to electromagnetic radiation, we sought to determine whether there is a disruption of the DNA methylation patterns in GC-2 cells exposed to 50 Hz ELF-EMF exposure for 72 h compared with the sham-exposure group. Quantitative analysis of the global DNA methylation levels showed that the DNA methylation in GC-2 cells were lower than the sham-exposure group at magnetic field intensity of 1 mT and were higher than the sham-exposure group at magnetic intensity of 2 mT and 3 mT (Figure 1). These data showed that the DNA in GC-2 cells acquired aberrant methylation pattern exposed to 50 Hz ELF-EMF exposure. It suggested that global methylation alterations might play a vital role in the biological effects and damage mechanism of 50 Hz ELF-EMF exposure.

### 3.2. Effect of 50 Hz ELF-EMF on the mRNA Expression of DNMTs

**Figure 2:**
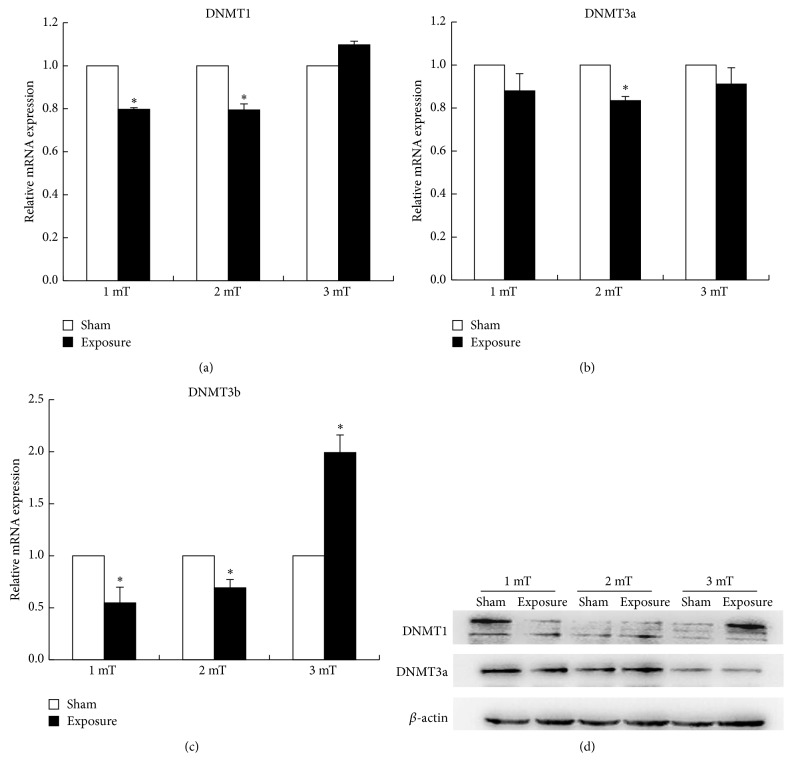
Effect of ELF-EMF electromagnetic field exposures on the mRNA and protein of DNMT1, DNMT3a, and DNMT3b in GC-2 cells. (a) The expression of DNMT1 was significantly lower than the sham-exposure group at magnetic field intensity of 1 mT and 2 mT and was significantly higher than the sham-exposure group at 3 mT. (b) The expression of DNMT3a decreased exposure to 50 Hz ELF-EMF exposure compared with the sham-exposure group at magnetic field intensity of 2 mT. (c) DNMT3b expression was significantly lower than the sham-exposure group at magnetic intensity of 1 mT and 2 mT and significantly higher than the sham-exposure group at 3 mT. (d) The protein expression of DNMT1 decreased at magnetic field intensity of 1 mT and increased at 3 mT.

As DNMTs play a crucial role in the regulation of DNA methylation pattern, we determined the expression profiles of DNMT1, DNMT3a, and DNMT3b. The mRNA expression of DNMT1, DNMT3a, and DNMT3b was measured by real-time PCR. As shown in Figures 2(a) and 2(c), the expression of DNMT1 and DNMT3b were significantly lower than the sham-exposure group at magnetic intensity of 1 mT and 2 mT and were significantly higher than the sham-exposure group at 3 mT. However, the expression of DNMT3a decreased exposed to 50 Hz ELF-EMF exposure compared with the sham-exposure group at 2 mT (Figure 2(b)).

### 3.3. Effect of 50 Hz ELF-EMF on the Protein Expression of DNMTs

We observed the differential mRNA expression of the DNMTs in the GC-2 cells exposed to ELF-EMF, and then we examined the protein expression of DNMTs in the GC-2 cells exposed to 50 Hz ELF-EMF exposure. We found that the protein expression of DNMT1 decreased at magnetic field intensity of 1 mT and increased at 3 mT electromagnetic field, while there were no obvious changes in DNMT3a expression between the exposure and sham group (Figure 2(d)). Unfortunately, DNMT3b expression was under the detection level (data not shown). These results were consistent with the quantitative analysis of the mRNA expression of the DNMTs using real-time PCR.

### 3.4. Differentially Methylated DNA Analysis in 50 Hz ELF-EMF Exposure

**Figure 3:**
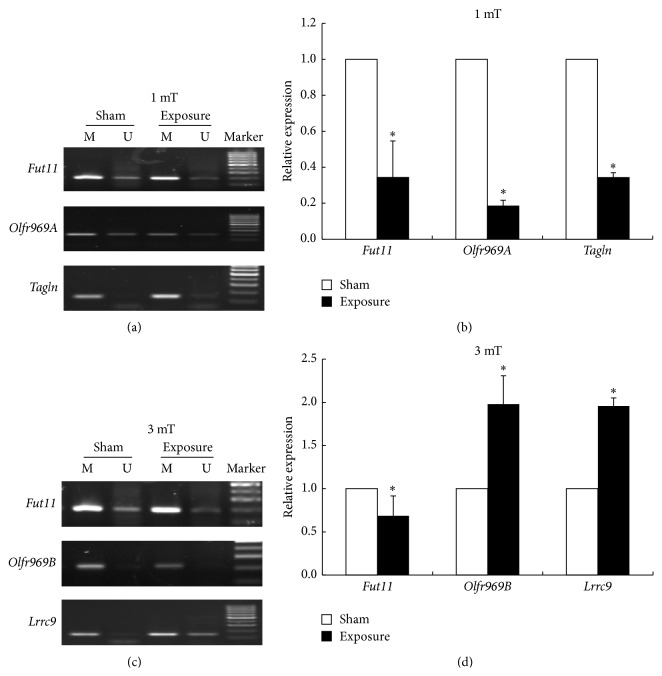
Differentially methylated genes verified with methylation-specific PCR and real-time PCR in 50 Hz ELF-EMF exposure. (a) Representative MSP results of the three genes (*Fut11*,* Olfr969A*, and* Tagln*) methylation in GC-2 cells at magnetic field intensity of 1.0 mT. M: methylated primers; U: unmethylated primers. (b) Validation of mRNA expression of the three genes (*Fut11*,* Olfr969A*, and* Tagln*) by real-time PCR. (c) Representative MSP results of the three genes (*Fut11*,* Olfr969B*, and* Lrrc9*) methylation in GC-2 cells at magnetic intensity of 3.0 mT. (d) Validation of mRNA expression of the three genes (*Fut11*,* Olfr969B*, and* Lrrc9*) by real-time PCR.

In order to investigate the potential mechanism, we screened differential methylated sites in detail using Affymetrix Mouse promoter 1.0R Array. Genome-wide methylation profiling across 50 Hz ELF-EMF exposure and control group were shown in Figure 3. Array analysis revealed that thousands of gene acquired aberrant methylation exposed to 50 Hz ELF-EMF exposure (fold change > 2). Through DNA methylation chip analysis, there were a total of 296 differentially methylated sites (including 166 hypermethylation and 130 hypomethylation) in the 1 mT exposed group compared with the control group. In 3 mT group, there were only 70 differentially methylated sites, including 11 hypermethylated and 59 hypomethylated sites. The methylation status of differential methylation sites was confirmed by methylation-specific PCR and the mRNA expression of genes was validated by real-time PCR in Figure 3. As shown in Figure 3(a), we found that* Fut11*,* Olfr969A*, and* Tagln* showed hypermethylation in GC-2 cells at magnetic field intensity of 1.0 mT. The mRNA expression of those three genes was downregulated at 1.0 mT as shown in Figure 3(b). At the same time,* Fut11* was hypermethylated, whereas* Olfr969B* and* Lrrc9* were hypomethylated in GC-2 cells at magnetic intensity of 3.0 mT as shown in Figure 3(c). The mRNA expression of* Fut11* showed downregulation and the expression level of* Olfr969B* and* Lrrc9* was upregulated in GC-2 cells at magnetic field intensity of 3.0 mT (Figure 3(d)). These results suggest that the methylation status of these genes was inversely correlated with the mRNA expression in GC-2 cells exposed to 50 Hz ELF-EMF.

### 3.5. Gene Expression Profiling in 50 Hz ELF-EMF Exposure

**Figure 4:**
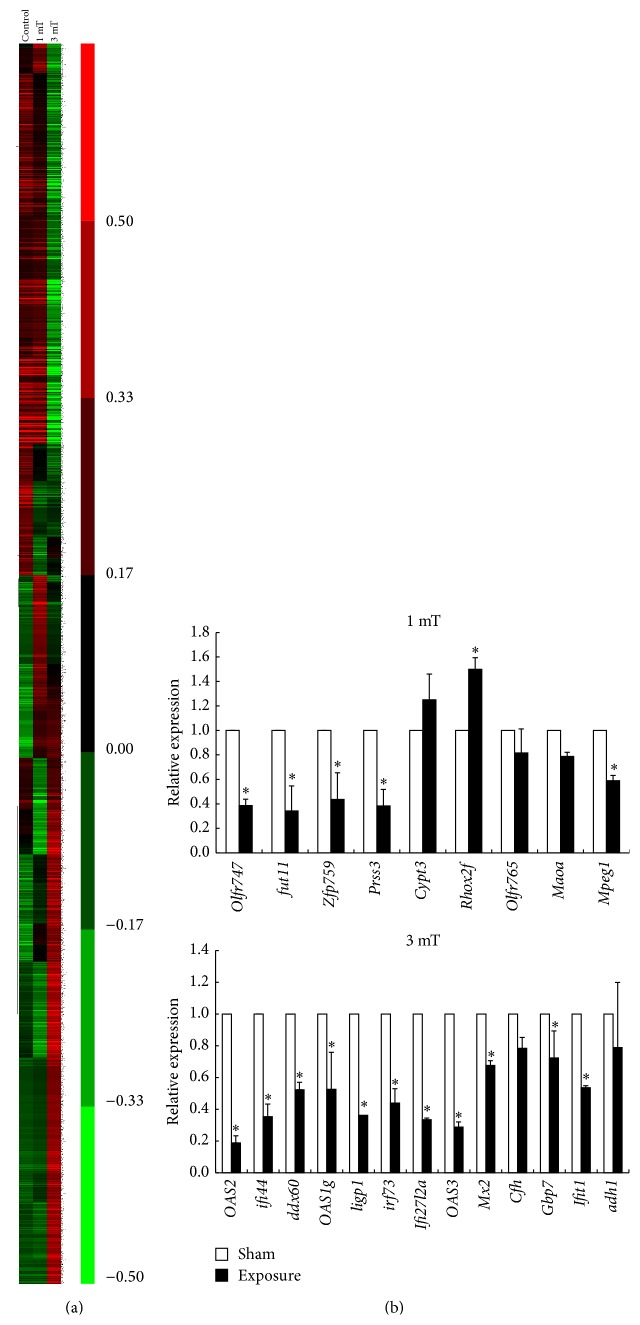
Clustering of differentially expressed genes across 50 Hz ELF-EMF exposure and control group (a) and the differentially regulated genes in GC-2 cells at magnetic field intensity of 1 mT and 3 mT were validated by real-time PCR (b). Gene expression data are presented in a matrix format. Each row represents an individual gene, and each column corresponds to an exposure group, with red indicating upregulation and green indicating downregulation. Black and gray indicate unchanged expression and missing value, respectively.

To further explore the effects of 50 Hz ELF-EMF, we used Affymetrix microarrays analysis to establish the gene expression profiles. Clustering of differentially expressed genes across 50 Hz ELF-EMF exposure and control group were shown in Figure 4(a). To investigate the potential mechanism, we choose the fold change that was greater than 1.5 as significantly differentially regulated genes at magnetic field intensity of 1 mT and 3 mT. Through gene expression chip analysis, there were a total of 84 differentially expression genes (including 44 genes upregulation and 40 downregulation) in the 1 mT exposed group compared with the control group. In 3 mT, the altered genes are 324, including 235 increase and 89 decrease. According to the results of Affymetrix Array, we chose several differentially regulated genes which were downregulated or upregulated in GC-2 cells at magnetic field intensity of 1 mT and 3 mT to test and verify using real-time PCR. These results showed that the mRNA expression of selected gene was consistent with the Affymetrix Array in GC-2 cells (Figure 4(b)).

Through pathway analysis, we found that 30 genes in Olfactory transduction pathway, 6 genes in Ribosome pathway, and 4 genes in Steroid hormone biosynthesis pathway upregulated, while 30 genes in Olfactory transduction pathway downregulated in the 1 mT exposed group compared with the control group. However, more genes and more pathways altered in 3 mT exposed group, including 21 genes in Ribosome pathway, 51 genes in Olfactory transduction pathway, 10 genes in Systemic lupus erythematosus pathway, 7 genes in p53 signaling pathway, 5 genes in Circadian rhythm pathway, 8 genes in Cytosolic DNA-sensing pathway, 9 genes in Drug metabolism pathway, 10 genes in Antigen processing and presentation pathway, 8 genes in Retinol metabolism pathway, and 17 genes in Cytokine-cytokine receptor interaction pathway.

### 3.6. Network Analyses

**Figure 5:**
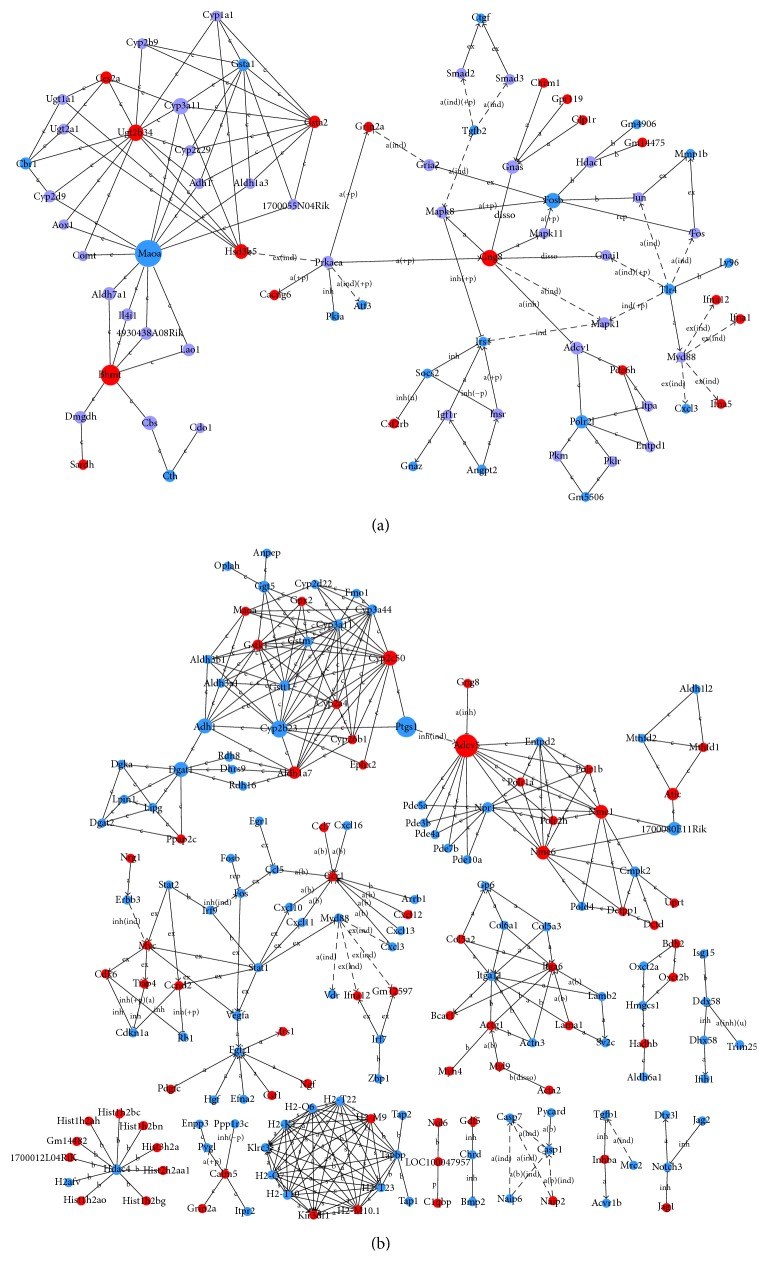
Network analysis of dynamic gene expression in GC-2 cells at magnetic field intensity of 1 mT (a) and 3 mT (b). The red dot stands for upregulated genes, the blue dot is downregulated genes, and the lilac dot stands for the connection gene.

To better elucidate the molecular mechanisms and biological pathways implicated in 50 Hz ELF-EMF exposure in GC-2 cells, network analyses were used to find the differentially expressed and target key genes in GC-2 cells between the exposure group and the sham-exposure group. Network analyses were generated through the use of the software confirmed the major functionally related gene groups. As seen from Figure 5(a),* Maoa*,* Bhmt*,* Gng8*,* Ugt2b34*,* Dgat1*, and* Adh1* may be target genes in GC-2 cells at magnetic intensity of 1 mT.* Cyp3a11*,* Ugt2b34*,* Adcy5*,* Ptgs1*, and* Cyp2b23* may be closely related with the epigenetic of 50 Hz ELF-EMF exposure at magnetic intensity of 3 mT (Figure 5(b)).

## 4. Discussion

In our study, we demonstrated for the first time that 50 Hz ELF-EMF exposure can induce the alterations of genome-wide methylation and the expression of* DNMTs* in spermatocyte-derived GC-2 cells. That is, the epigenetics might play an important role in the biological effects of 50 Hz ELF-EMF exposure.

Several studies have found reproductive toxicity of EMFs in male [26, 27]. In male rats, 50 Hz sinusoidal magnetic field at approximately 25 mT for 18 consecutive weeks did not have effects on the weight of the testes but reduced significantly the weights of seminal vesicles and preputial glands and sperm count [26]. Continuous exposure of 50 Hz ELF-EMF of 0.1 or 0.5 mT for 8 weeks significantly increased the incidence of testicular germ cell death [28]. The similar results were repeated in mice by 16-week continuous exposure to 60 Hz MF of 14 mT [10]. In addition, 50 Hz ELF EMF induced alterations of spermatozoa motility and kindling rate in rabbits, therefore influencing fertility [13]. At the cellular level, Li et al. found that short-term but not long-term ELF-EMF exposure may decrease the reproductive ability of male* Drosophila melanogaster* through the caspase pathway mediated spermatogenesis [29]. The increase of free radicals and Ca^2+^ levels may lead to the ELF-EMF induced toxicity [11, 30]. In addition, ROS may be involved in cell growth inhibition by apoptosis and arrested the cell cycle in 60 Hz ELF-EMF exposed prostate cancer cells [31]. However, the molecular mechanism is still unclear.

Recently, DNA methylation via the regulation of chromatin structure modifications and the expression of genes involved in cell cycle checkpoints, apoptosis, and DNA repair is closely related to embryonic development, autoimmune diseases, cancer, and central nervous system diseases [31]. Therefore, we speculated that DNA methylation might be associated with 50 Hz ELF-EMF exposure. First, we detected genome-wide methylation status of GC-2 cell line exposed to 50 Hz ELF-EMF at magnetic field intensity of 1 mT, 2 mT, and 3 mT with an intermittent exposure for 72 h by Methylated DNA Quantification Kit. To our knowledge, this is the first report on the status of global DNA methylation in GC-2 cells subjected to 50 Hz ELF-EMF exposure. We found that genome-wide methylation in GC-2 cells decreased at magnetic intensity of 1 mT and increased at 3 mT exposure to 50 Hz ELF-EMF compared with the control. Taken together, these data suggest that DNA hypomethylation may be an important epigenetic event in the process of 50 Hz ELF-EMF exposure on GC-2 cells. Although our results need to be confirmed in animal and human study, it appears that there is a direct relationship between DNA hypomethylation and 50 Hz ELF-EMF exposure.

Then, we applied real-time PCR and Western bolt to detect the mRNA and protein expression of DNMT1, DNMT3a, and DNMT3b, respectively. We observed that DNMT1 and DNMT3b decreased at magnetic field intensity of 1 mT and increased at 3 mT. However, the expression of DNMT3a had no obvious change exposure to 50 Hz ELF-EMF exposure. DNMT1 is involved in the maintenance of methylation, while DNMT3 is involved in de novo methylation. Thus, the alterative expression of DNMTs might contribute to the alterations of genome-wide methylation in GC-2 cells.

In order to further investigate the potential mechanism, Affymetrix microarray was used to screen differential methylated sites in detail. The results of microarrays were confirmed using real-time PCR. Through differential signal pathway analysis, we found that the most obvious change in expression is olfactory transduction pathway. Olfactory receptor (OR) genes, mainly expressed in the surface of neurons in the olfactory epithelium, were also found in other tissues such as testis, heart, lung, kidney, and brain [32]. Genome analysis showed that about 5% to 10% of the olfactory receptor gene were expressed in late spermatocytes and early and late round sperm cells of testes in rodents [33, 34]. Fukuda et al. found that the sperm chemotaxis changed significantly in mice with higher expression of human OR17-4 [35]. These studies suggest that olfactory receptor gene may be looking to provide the guiding role of the egg cell or sperm selection. Thus, olfactory transduction pathway may play an important role in the biological effect of 50 Hz ELF-EMF.

Previous studies showed that immune cell activation may have important role in biological effect of 50 Hz ELF-EMF [36–39]. In this study, we also found that the expression of several immune-related gene has changed significantly. 2′,5′-Oligoadenylate synthetize genes (*OAS1*,* OAS2*,* and OAS3*) encode a family of enzymes pivotal to innate antiviral defense. Oligoadenylate synthetases (*OAS*) play an important role in the immune response against dengue virus. SNPs in the* OAS* genes are known to affect* OAS* activity and are associated with outcome of viral infections [40, 41]. In addition, the gene* Mx2* encodes a member of the Mx protein family of large GTPases and functions in the innate immunity system. Interferon alpha/beta treatment or viral infection induces expression of this protein, which subsequently accumulates in the cytoplasm and inhibits viral replication [42–44].* Ddx58* gene, also known as* RIG-I*, can sense viral RNAs and triggers innate antiviral responses through induction of type I IFNs and inflammatory cytokines. It has been reported that Rig-I functions as a positive regulator for NF-*κ*B signaling and is involved in multiple biological processes in addition to host antivirus immunity [45, 46].

In conclusion, we demonstrated that 50 Hz ELF-EMF exposure can induce the alterations of genome-wide methylation and the expression of DNMTs. These results suggest that epigenetic regulation might play an important role in the biological effects of 50 Hz ELF-EMF exposure. Although we could not confirm whether the long-term exposure of alternating current does harm to our health based on the present study, we recommend that charge power supply and high voltage wire should be kept as far as possible from our body to reduce the absorption of radiation by cells.

## Supplementary Material

Table S1: Primer sequences used for MSP in this study.
