# Supplementary material for: Effect of 50 Hz Extremely Low-Frequency Electromagnetic Fields on the DNA Methylation and DNA Methyltransferases in Mouse Spermatocyte-Derived Cell Line GC-2
Source: Biomed Res Int. 2015 Aug 3;2015:237183. doi: 10.1155/2015/237183 (PMC4538330; doi:10.1155/2015/237183)
Supplement: Supplementary file 1 — Table S1: Primer sequences used for MSP in this study. [file 237183.f1.doc]

**Table S1** Primer sequences used for MSP in this study

| Gene |  | Primer sequence(5’-3’) | Length  (bp) | Annealing temperature(℃) |
| --- | --- | --- | --- | --- |
| Sdpr | MSP(M) | Forward: TTATAGAATTTTGTTTTATGGT | 283 | 50 |
|  |  | Reverse: TTATCTAAACTAATTTAACCG |  |  |
|  | MSP(U) | Forward: TTATAGAATTTTGTTTTATGGT |  |  |
|  |  | Reverse: TTATCTAAACTAATTTAACCA |  |  |
| Tagln | MSP(M) | Forward: TTAGTTTGTTACGAGTTTGGC | 142 | 55 |
|  |  | Reverse: ACGACGAAAAAATCAAACTC |  |  |
|  | MSP(U) | Forward: AGGTTAGTTTGTTATGAGTTTGGT |  |  |
|  |  | Reverse: AAAACAACAAAAAAATCAAACTC |  |  |
| Lrrc9 | MSP(M) | Forward: ATTTTAGTAGACGTTGTGGTTGA | 159 | 55 |
|  |  | Reverse: AAAATTCTCAAACAAACTTTCG |  |  |
|  | MSP(U) | Forward: ATTTTAGTAGATGTTGTGGTTGA |  |  |
|  |  | Reverse: AAAATTCTCAAACAAACTTTCA |  |  |
| Fut11 | MSP(M) | Forward: GTTATTAGAATTGGTTTTGTTC | 203 | 55 |
|  |  | Reverse: CACTATTACTCAAACTATACCCA |  |  |
|  | MSP(U) | Forward: GTTATTAGAATTGGTTTTGTTT |  |  |
|  |  | Reverse: CACTATTACTCAAACTATACCCA |  |  |
| Olfr969A | MSP(M) | Forward: TATTCGAATGTAATTTGTTTC | 221 | 50 |
|  |  | Reverse: TCATTCAATAAAACTATCCAA |  |  |
|  | MSP(U) | Forward: TATTTGAATGTAATTTGTTTT |  |  |
|  |  | Reverse: TCATTCAATAAAACTATCCAA |  |  |
| Nod1 | MSP(M) | Forward: GTGGTTGGTTAGGTTATAGTC | 290 | 50 |
|  |  | Reverse: TAATAAAACAAAATAAATACATAA |  |  |
|  | MSP(U) | Forward: GTGGTTGGTTAGGTTATAGTT |  |  |
|  |  | Reverse: TAATAAAACAAAATAAATACATAA |  |  |
